# Healing of bilateral humeral intracondylar fissures following placement of locking 4.5 mm transcondylar screws

**DOI:** 10.1111/jsap.70032

**Published:** 2025-09-28

**Authors:** A. Belch, A. Bourbos, D. Carwardine

**Affiliations:** ^1^ Langford Vets Small Animal Referral Hospital Bristol UK

A 7‐month‐old entire male English springer spaniel presented with a two‐month history of bilateral forelimb lameness, worse on the left side. Clinical examination revealed bilateral elbow discomfort. A computed tomography (CT) scan was performed (Siemens Somatom Go‐Top, Siemens Healthineers, Erlangen, Germany), which revealed bilateral humeral intracondylar fissures, more severe on the left side (Fig 1A, B). Using fluoroscopic guidance, a minimally invasive approach was employed to place a 4.5 mm transcondylar locking screw (Arthrex GmbH, Munich, Germany) bilaterally (Fig 1E–H). The patient was discharged with a five‐day course of meloxicam and instructions for crate rest and short leash walks (10 minutes, three times daily) for 6 weeks. At 6 weeks postoperatively, the dog was reassessed; no lameness or elbow discomfort was noted, and elbow manipulation was comfortable. A repeat CT scan, performed using an extended scale (metal dampening) protocol, demonstrated stable implants and progressive healing of the intracondylar fissures (Fig 1C, D). Recent publications have advocated proximal oblique osteotomies or the application of bone grafts/demineralised bone matrix with compression to promote healing of humeral intracondylar fissures. This case demonstrates that, with appropriate case selection, a well‐placed positional screw can achieve healing without the need for additional interventions.

**FIG 1:**
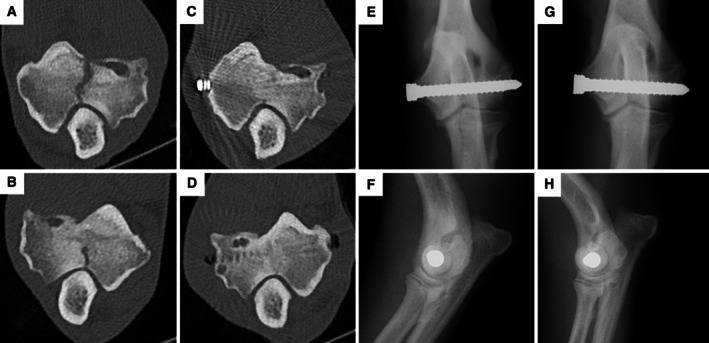
Transverse slice through intracondylar fissure in left (A) and right (B) elbows. Left (C) and right (D) elbow at 6 weeks postoperatively showing near complete healing of the fissure. Left (E, F) and right (G, H) immediate postoperative fluoroscopic views showing placed of a 4.5 mm transcondylar screw.

## Author contributions

**Alex Belch:** Writing – review and editing, conceptualization, writing – original draft. **Alexandros Bourbos:** Conceptualization. **Darren Carwardine:** Conceptualization, review and editing.

